# Explaining the perception and experiences of breastfeeding in mothers who have a high risk pregnancy: a protocol study

**DOI:** 10.1186/s12978-024-01817-x

**Published:** 2024-06-01

**Authors:** Kobra Mirzakhani, Atefeh Yas, Talat Khadivzadeh

**Affiliations:** 1 Nursing and Midwifery Care Research Center, Mashhad University of Medical Sciences, Mashhad, Iran; 2 Department of Midwifery, School of Nursing and Midwifery, Mashhad University of Medical Sciences, Mashhad, Iran; 3 Student Research Committee, Mashhad University of Medical Science, Mashhad, Iran

**Keywords:** Breastfeeding, Mothers, High-risk pregnancy, Content analysis, Qualitative study

## Abstract

**Introduction:**

Enhancing breastfeeding practices, even in affluent nations, significantly reduces child mortality rates. Nevertheless, three out of five newborns do not receive breastfeeding within the first hour of birth. Research indicates that under high-risk pregnancy circumstances, there may be challenges in initiating and sustaining breastfeeding. Infants born from high-risk pregnancies are particularly vulnerable to illnesses and mortality. Although breastfeeding serves as a protective measure against various infant and post-infancy ailments, many mothers encounter difficulties in commencing or maintaining breastfeeding due to complications associated with their conditions. The present study aims to illuminate the understanding and experience of breastfeeding in mothers with high-risk pregnancies, considering the cultural and social context of Iran.

**Method:**

This study is a qualitative research utilizing a conventional content analysis approach. In this qualitative study, mothers who have undergone a high-risk pregnancy and currently have infants under 6 months old will be chosen through purposeful and snowball sampling. Their breastfeeding experiences will be gathered through individual, semi-structured, and face-to-face interviews. In addition to interviews, observation and focus groups will also be used to collect data. Data analysis was performed using Graneheim and Lundman’s method with MAXQDA software version 10, VERBI Software GmbH, Berlin. The study will utilize the criteria of Lincoln and Guba (1985) for validity and reliability.

**Discussion:**

This qualitative study aims to investigate the experiences and challenges of breastfeeding in mothers with high-risk pregnancies to pinpoint breastfeeding barriers in this demographic and develop essential interventions and strategies to address these obstacles.

## Introduction

Breastfeeding is viewed as a distinctive method of nourishing infants, offering both short-term and long-term emotional, nutritional, and immunological advantages [1]. Boosting breastfeeding rates has the potential to save the lives of up to 800,000 children, even in high-income nations. It also contributes to maternal health, with estimates suggesting that breastfeeding prevents 20,000 deaths annually from breast cancer [2, 3]. However, statistics reveal that 3 out of every 5 infants are not breastfed within the first hour after birth, increasing their vulnerability to mortality and illness [4]. Breastfeeding not only enhances the well-being of both mother and child but is also acknowledge d as a key strategy for attaining sustainable development goals [5]. Aligned with global health and development targets, the World Health Organization pledged in 2012 to raise the worldwide rate of exclusive breastfeeding in the initial 6 months of life from 37 to 50% by 2025 by supporting breastfeeding initiatives [6]. A systematic review in Iran showed that the prevalence of exclusive breastfeeding varies from 61 to 82% [7].

Studies indicate that the presence of risk factors during pregnancy can delay the initiation and continuation of breastfeeding, leading to reduced success for mothers [8]. Cordero et al. (2016) found that mothers with diabetes, substance abuse, and preterm labor have lower rates of breastfeeding initiation compared to healthy women [9]. Similarly, Scime et al. [10] demonstrated that prenatal medical risk is linked to shorter breastfeeding duration. Maternal obesity, a significant risk factor in high-risk pregnancies, is associated with various breastfeeding challenges, reduced initiation, and shorter exclusive breastfeeding duration [11, 12]. High-risk pregnant mothers also experience physical and emotional distress due to health concerns, anxiety, and worries about their newborns [13]. Additionally, there are concerns about the impact of medication use on the quality of breast milk, which can disrupt maternal roles in this group of mothers [14, 15]. Therefore, efforts to promote breastfeeding among mothers who have experienced high-risk pregnancies are essential [16].

In some cases, breastfeeding in high-risk pregnancies can have positive effects on maternal health, such as improving left ventricular function and maternal outcomes in diseases like peripartum cardiomyopathy (PPCM) [17, 18]. Long-term breastfeeding in mothers with gestational diabetes can also reduce the risk of maternal diabetes postpartum [19]. Breastfeeding in mothers with gestational hypertension is associated with lower blood pressure in postpartum [20].

Research has focused on factors influencing the initiation and continuation of breastfeeding in high-risk pregnant women. Some evidence suggests that parents have the most influence on deciding to start or stop breastfeeding. Official support may also impact breastfeeding practices. Family and friends play a role in encouraging breastfeeding, and environmental support can be crucial [8, 21, 22]. Support in breastfeeding is vital in preventing maternal fatigue and increasing cardiac workload, especially in high-risk pregnancies such as congenital heart diseases (CDH) [15]. Integration of breastfeeding education and counseling in the care of high-risk pregnant women is necessary to address their knowledge and skill gaps [9]. Educational and supportive programs can prevent delays in breastfeeding initiation in women with preeclampsia [23].

Social-cultural factors such as a mother’s beliefs and those of her surroundings about infant nutrition are influential factors in exclusive breastfeeding [8]. Breastfeeding until the age of two has been emphasized in the Holy Quran. In Iran, it seems that breastfeeding is rooted more in Iranian and Islamic culture dominating society than in scientific advancements [7, 16]. On the other hand, healthcare providers need to be aware of the determinants of exclusive breastfeeding with maternal milk to offer preventive solutions to overcome these barriers and help improve the health outcomes of mothers and children [8].

Improving breastfeeding rates among all mothers, with a focus on groups of the population with the lowest breastfeeding rates, is a key objective of the health sector. Comprehensive interventions involving hospitals, culture, and community are necessary to improve breastfeeding-related indicators [16, 24].

Pregnant mothers in high-risk populations face various health consequences and risks. Therefore, understanding their perspective on breastfeeding is crucial for effective interventions. Exploring the breastfeeding experiences of these mothers, identifying barriers and facilitators, can offer valuable insights and guide midwives in designing interventions to enhance maternal and infant health and reduce health disparities. Due to the lack of comprehensive research on the breastfeeding experiences of at-risk pregnant mothers, it is essential to investigate their perceptions of initiating and sustaining breastfeeding and the support they require within the cultural and social context of Iranian society. Hence, a qualitative study has been planned to uncover the experiences of these mothers and develop necessary solutions and interventions to promote successful breastfeeding among high-risk pregnant mothers. Utilizing qualitative research with conventional content analysis methodology is recommended for its ability to elucidate the understanding and experiences of concepts, enabling researchers to address their research inquiries effectively. This approach allows researchers to interpret data authentically and scientifically [25, 26].

### Main objective

To elucidate the understanding and experience of breastfeeding in mothers with high-risk pregnancies.

### Specific objectives

- Clarifying the understanding of breastfeeding in mothers with high-risk pregnancies.
- Elucidating the experience of breastfeeding in mothers with high-risk pregnancies.
- Clarification of health care workers’ understanding of breastfeeding in mothers with high-risk pregnancies.

### Primary research question

What is the understanding and experience of breastfeeding in mothers with high-risk pregnancies?

## Method

The present study is a qualitative research using a conventional content analysis approach. Qualitative content analysis is a flexible method for analyzing various types of data and information. It can be considered as a research method for interpreting the textual data, involving coding, identifying themes, or designing patterns. This approach aims to condense data, organize data, and develop theories and models. Qualitative content analysis is also applicable in cases where the study aims to achieve a comprehensive and extensive description of a phenomenon, introduce reality, create a new overall perspective or concepts, or create descriptive categories that describe the phenomenon [25].

Considering the researcher’s intention to comprehensively describe and explain mothers’ understanding and experience after a high-risk pregnancy regarding breastfeeding and their decision-making process for breastfeeding, and considering the sensitivity and cultural aspects of breastfeeding, conventional content analysis appears to be the most appropriate approach for this study.

### Inclusion and exclusion criteria

Inclusion criteria include mothers aged 15 to 49 years with a baby under 6 months old, having at least one risk factor during pregnancy such as gestational diabetes, hypertension during pregnancy, BMI over 29, treated mental illness, bleeding during pregnancy, infection, premature birth, internal surgical diseases during pregnancy (e.g., heart, kidney, gastrointestinal diseases), having at least one attempt by the mother to breastfeed and speak in Persian. The exclusion criteria also includes the mother’s unwillingness to participate in the interview.

### Setting and sampling

The research environment includes teaching hospitals and healthcare centers in the city of Mashhad. These environments are chosen for easy access and maximum participant diversity. Sampling will be purposeful and based on the snowball method to ensure maximum diversity.

Researchers will commence sampling after obtaining approval from the ethics committee of Mashhad University of Medical Sciences and obtaining a referral letter from the School of Nursing and Midwifery in Mashhad. They will initiate sampling by visiting the research environment officials, providing explanations and outlining the research objectives.

Researchers will aim to ensure maximum diversity in the samples concerning age, infant age, type of baby feeding, socio-economic status, type of pregnancy-related condition, and healthcare interventions. Using the snowball method, employed midwives in health centers will be asked to connect researchers with mothers knowledgeable about the research topic. Subsequently, researchers will contact selected participants via phone, explain the research goals, invite them to join the study, and interview interested mothers who meet the study criteria. Participants will receive necessary information about voluntary participation, confidentiality, and anonymity. The interviews will be conducted by two trained researchers who will establish rapport to gain participants’ trust and obtain written consent. Through semi-structured individual interviews and face-to-face interactions, researchers will explore participants’ emotions, perceptions, and thoughts.

### Data collection

An interview with an open-ended question with mothers about their experience of initiating and sustaining breastfeeding will continue with exploratory questions to provide further explanation. An interview guide has been prepared for this purpose (Talk about your experience of starting and continuing breastfeeding, Talk about your experience about deciding to start breastfeeding, Talk about your experience about deciding to continue breastfeeding, Express your understanding of the influence of formal and informal support networks on the decision and continuation of breastfeeding).

During the interview, participants will be encouraged to describe their experiences regarding the initiation and continuation of breastfeeding to clarify the decision-making process and to express the obstacles and facilitating factors that have influenced their decision and ability to breastfeed or reduce and stop it. Participants’ understanding of the impact of their formal and informal support networks on their decisions regarding breastfeeding will also be examined. At the end of the interviews, participants will be asked to take note of any additional experiences or insights that come to mind later and provide them to the researcher. Following expressions of gratitude and appreciation, participants will be informed about the possibility of future face-to-face or remote interviews, and a suitable gift will be provided at the end of the session as a token of appreciation.

The duration of the interviews is estimated to be between 60–90 min, depending on the amount of information and participants’ circumstances. With participants’ permission, the interviews will be fully recorded using an MP3 player. The interviews will take place in a quiet room with adequate facilities for the comfort of breastfeeding mothers, considering the conditions of healthcare centers, homes, or any other location desired by the breastfeeding mother. Sampling will continue until no new information is obtained from the interviews and until data saturation is reached [27].

The breastfeeding behavior of mothers and communication between mothers and health care workers for breastfeeding will be observed in the hospital after the birth of the baby. A trained qualitative researcher (AY) will carry out the non-participant observations, a method in which the researcher does not participate actively, but just observes a situation without interfering. The observation takes place in natural settings, allowing the observer to evaluate non-verbal cues, thus increasing ecological validity. Prior to requesting consent for the observation, the researcher will clarify that she will be observing the entire session, taking notes, and recording the conversation with a smartphone.

A focus group will be used to explain the health providers’ understanding of breastfeeding in mothers with high-risk pregnancies. Health providers (including midwives, nurses and gynecologists and pediatricians) working in women’s hospital and health centers (with work experience of at least 5 years) will participate in the sessions. It is expected that 3–4 sessions will be held. Approximately 8–12 health care workers will be present in each session. The aims of the focus group will be explained, and consent will be sought for audio recording. The focus group will last approximately 2 h. Researchers will conduct the focus group, one observe and take notes of the discussion and two will conduct the conversation guided by a list of topics prepared prior to the group (How is breastfeeding in mothers with high-risk pregnancies? What factors affect the breastfeeding of this group of mothers?). The focus group’s audio recording will be transcribed verbatim. Then, the researchers team will read the transcripts separately and analysis the data.

### Data analysis

Data analysis will be conducted simultaneously with data collection. MAXQDA software version 10, developed by VERBI Software GmbH in Berlin, will be used for managing and organizing the data.

Data analysis was conducted using Graneheim and Lundman’s method in four stages [28]. Stage 1 involved reading the interviews multiple times to grasp their content and gain insights by exploring both latent and manifest content. In Stage 2, the text of each interview transcript was divided into meaningful units such as words, sentences, and paragraphs, where connections between different texts were identified. Stage 3 focused on condensing the meaningful units and assigning codes to them. In Stage 4, the codes were compared for similarities and differences, with similar codes grouped into initial categories. As the analysis progressed, the initial categories were refined, and subcategories were formed. Finally, main categories emerged from the integration of similar subcategories.

### Validity and reliability of the study

In the present research, the validity and reliability of the findings will be evaluated using criteria established by Lincoln and Guba (1985). The researchers have suggested four indicators to bolster the credibility of qualitative research: credibility, dependability, confirmability, and transferability [29].

To enhance the credibility and acceptance of the data, the researcher aims to offer recorded information and codes to the participants throughout the study to validate the main themes derived from mothers’ accounts of breastfeeding experiences, barriers to initiation, and factors supporting breastfeeding continuation. In this research, information and data are gathered from participants with diverse experiences. Sufficient data collection, selection of appropriate meaningful units, description of category and theme development, explanation of how judgments are made regarding similarities and differences between categories through the use of participants’ quotes all contribute to the credibility of the data.

Researchers will aim to establish effective communication with participants. Additionally, to boost data credibility, all interviews will be recorded verbatim and transcribed word by word. Throughout the research write-up, participants’ statements will be accurately documented, and data will be meticulously maintained. The researcher will clarify how units of meaning are summarized and condensed, how categories are formed, and how themes are developed. The research process and decisions taken along this journey will be reported to facilitate follow-up by other researchers. To ensure auditability, activities will be documented over time so that others can trace the subject by reading these writings. For the transferability of findings, the researcher will offer a detailed description of the research context, participant involvement, sampling methods, and data collection time and location to confirm data transferability for readers.

## Discussion

The prevalence of high-risk pregnancy in Iran has been reported to be 52–8/39% [30, 31], and the rate of exclusive breastfeeding in Iran up to 6 months is 53% [32]. Studies indicate that in high-risk pregnancy conditions or the occurrence of pregnancy risk factors, there is a possibility of reduced success in breastfeeding. While infants born from high-risk pregnancies are at a higher risk of disease and mortality, breastfeeding protects them against many infant and post-infancy diseases. Therefore, identifying facilitators and barriers to breastfeeding in mothers with high-risk pregnancies is of particular importance, and the current research also helps in discovering and explaining the obstacles to breastfeeding in this group of mothers so that by implementing specific actions and interventions to address them, it can contribute to improving breastfeeding indicators.

## Data Availability

No datasets were generated or analysed during the current study.
